# Pharmacological Doses of Daily Ascorbate Protect Tumors from Radiation Damage after a Single Dose of Radiation in an Intracranial Mouse Glioma Model

**DOI:** 10.3389/fonc.2014.00356

**Published:** 2014-12-15

**Authors:** Carole Grasso, Marie-Sophie Fabre, Sarah V. Collis, M. Leticia Castro, Cameron S. Field, Nanette Schleich, Melanie J. McConnell, Patries M. Herst

**Affiliations:** ^1^Malaghan Institute of Medical Research, Wellington, New Zealand; ^2^School of Biological Sciences, Victoria University, Wellington, New Zealand; ^3^Department of Radiation Therapy, University of Otago, Wellington, New Zealand

**Keywords:** pharmacological ascorbate, radiation, intracranial mouse glioma model, GL261, radioprotection, radiosensitization

## Abstract

Pharmacological ascorbate is currently used as an anti-cancer treatment, potentially in combination with radiation therapy, by integrative medicine practitioners. In the acidic, metal-rich tumor environment, ascorbate acts as a pro-oxidant, with a mode of action similar to that of ionizing radiation; both treatments kill cells predominantly by free radical-mediated DNA damage. The brain tumor, glioblastoma multiforme (GBM), is very resistant to radiation; radiosensitizing GBM cells will improve survival of GBM patients. Here, we demonstrate that a single fraction (6 Gy) of radiation combined with a 1 h exposure to ascorbate (5 mM) sensitized murine glioma GL261 cells to radiation in survival and colony-forming assays *in vitro*. In addition, we report the effect of a single fraction (4.5 Gy) of whole brain radiation combined with daily intraperitoneal injections of ascorbate (1 mg/kg) in an intracranial GL261 glioma mouse model. Tumor-bearing C57BL/6 mice were divided into four groups: one group received a single dose of 4.5 Gy to the brain 8 days after tumor implantation, a second group received daily intraperitoneal injections of ascorbate (day 8–45) after implantation, a third group received both treatments and a fourth control group received no treatment. While radiation delayed tumor progression, intraperitoneal ascorbate alone had no effect on tumor progression. Tumor progression was faster in tumor-bearing mice treated with radiation and daily ascorbate than in those treated with radiation alone. Histological analysis showed less necrosis in tumors treated with both radiation and ascorbate, consistent with a radio-protective effect of ascorbate *in vivo*. Discrepancies between our *in vitro* and *in vivo* results may be explained by differences in the tumor microenvironment, which determines whether ascorbate remains outside the cell, acting as a pro-oxidant, or whether it enters the cells and acts as an anti-oxidant.

## Introduction

The use of high (pharmacological) doses of ascorbate for the treatment of cancer is highly controversial [reviewed by Ref. (1)]. Although most mammals make their own ascorbate from glucose in the liver, humans and other primates (as well as guinea pigs and bats) obtain ascorbate from their diet as they lack a functional gulonolactone oxidase necessary for the last step of ascorbate synthesis (2). Regardless of source, systemic ascorbate levels are very tightly regulated by controlled uptake into (1) the blood stream through the intestinal epithelium (via high affinity sodium-dependent vitamin C transporters, SVCT1) and (2) into body tissues (via SVCT2), followed by (3) renal clearance (via SVCT1) to plateau at 40–60 μM (3). Intravenous/intraperitoneal injection of high-dose ascorbate (0.5–1.25 g/kg in humans; 1–4 g/kg in mice) can bypass regulation to generate serum levels of 30 mM for about 2 h (4, 5). Such high concentrations of ascorbate have been reported to generate extracellular hydrogen peroxide, particularly in the acidic metal-rich environment of solid tumors [reviewed by Ref. (1)]. Hydrogen peroxide diffuses into cells where it generates reactive oxygen species (ROS), such as hydroxyl radicals (6), which overwhelm the anti-oxidant defense system, resulting in DNA damage (7, 8). The extracellular addition of catalase abrogates the pro-oxidant effect of high-dose ascorbate (6–9), verifying the role of extracellular hydrogen peroxide in its mechanism of action.

High-dose ascorbate is well tolerated in cancer patients who have normal renal function, do not have glucose-6-phosphate dehydrogenase deficiency or iron overload (4, 5) and has been shown to delay tumor growth in rodent xenograft models (10). However, high-dose ascorbate as a single agent has never been reported to cure cancer in animals or humans. Recent preclinical research has therefore focused on combining high-dose ascorbate with chemotoxic anticancer drugs such as Carboplatin and Paclitaxel for ovarian cancer (11), Gemcitabine for pancreatic cancer (12), Epigallocatechin-3-gallate plus Gemcitabine for malignant mesothelioma (13), Vincristine (14), and glycolytic inhibitors for small cell lung cancer (15). Potentiation of the hydrogen peroxide generating effect of ascorbate *in vitro* has been reported for inhibitors of hydrogen peroxide metabolism in pancreatic cancer (16), alpha-tocopherol succinate in prostate cancer (17), manganoporphyrins in breast cancer (18) and pancreatic cancer (19), and phenylaminonaphthoquinones in bladder cancer (20). Increased sensitivity to Docetaxel, Epirubicin, Irinotecan, and 5-FU after ascorbate exposure has been attributed to G0/G1 arrest in prostate carcinoma cells (21).

Only three small clinical trials have so far been published that have evaluated the effect of high-dose ascorbate combination therapy in metastatic ovarian cancer [with carboplatin plus paclitaxel; *n* = 25 (11)] and metastatic pancreatic cancer [with gemcitabine; *n* = 9 (22) and with gemcitabine plus erlotinib; *n* = 14 (23)]. All three trials noted that patients on the ascorbate plus chemotherapy arm lived longer than those taking conventional chemotherapy alone. However, the increase in survival time was not statistically significant due to the small patient numbers typical of stage I trials (11, 22, 23). With respect to side-effects, all trials demonstrated a lack of ascorbate-related toxicities. In fact, two trials (11, 22) reported fewer grade 1 and 2 chemotherapy-related toxicities with the addition of ascorbate to standard chemotherapy.

Radiation resistance of the primary adult brain cancer, glioblastoma multiforme (GBM), is a key factor in poor patient outcomes (24). As the blood–brain barrier limits chemotherapy options for brain tumors radiation is a primary treatment. Patient outcomes can be improved by either escalation of radiation dose to the tumor (25) or by increasing the sensitivity of GBMs to radiation. Because ionizing radiation kills cells mainly by generating free radicals that cause double stranded DNA breaks (DSB) (26), one approach to radio-sensitization would be to combine radiation therapy with compounds that also generate free radicals. As many cancers have inferior radical scavenging and DNA repair capability (27), a combination of treatments that increase free radical production should theoretically affect cancer cells more than normal cells.

Two *in vitro* studies have shown that high-dose ascorbate radiosensitized primary GBM cells isolated from tumors of GBM patients by generating extracellular hydrogen peroxide and inducing S/G2 arrest, interfering with DNA repair (7, 8). Interestingly, both ionizing radiation and high-dose ascorbate were shown to increase labile iron levels in pancreatic tumor homogenates from athymic nude mice (28). Catalytic metals can accelerate ascorbate oxidation, leading to increased generation of hydrogen peroxide (29). Radio-sensitization of highly aggressive radio-resistant GBMs has significant clinical implications. The addition of ascorbate to radiation protocols would allow for superior tumor control at lower radiation doses, resulting in less severe acute and chronic brain toxicities. The first report of ascorbate chemoradiation in mice was published in 1996 when Taper and colleagues showed that pretreating intramuscularly transplanted liver tumors with ascorbate and menadione (Vitamin K_3_) potentiated the effect of a single dose of 20–50 Gy of X-ray irradiation (30). Here, we investigate the effects of high-dose ascorbate and radiation on tumor progression and tumor histology in an intracranial mouse glioma model, where GL261 mouse glioma cells are injected directly into the brain of immunocompetent C57BL/6 mice.

## Materials and Methods

### Materials

Unless otherwise noted, tissue plasticware was purchased from Nunc (ThermoFisher Scientific, Auckland, New Zealand); all cell culture reagents were from Gibco BRL (Invitrogen, Auckland, New Zealand). Sodium ascorbate and all other chemicals and reagents were from Sigma Chemical Company (St. Louis, MO, USA).

### Cell lines

The mouse glioma cell line, GL261 was obtained from the NCI tumor cell line repository (Fredrick, MD). GL261 cells were grown in RPMI-1640 supplemented with 20% (v/v) FBS, GlutaMAX-1 (2 mM), 25 μg/mL penicillin, 25 μg/mL streptomycin and maintained in a humidified incubator at 37°C/5% CO_2_.

### Irradiation of GL261 cells

GL261 cells were seeded 24 h prior to treatment in six well plates (3 to 5 × 10^4^/well). Cells were 30–40% confluent and growing exponentially on the day of treatment. Cells were irradiated with 1, 3, 6, or 9 Gy using Cesium-137 γ-rays (Gammacell 3000 Elan, Best Theratronics) for viability assays and with 6 Gy for clonogenicity assays. After irradiation, the cells were re-incubated in fresh medium.

### Ascorbate treatment of GL261 cells

Exponentially growing 30–40% confluent GL261 cells were seeded 24 h prior to treatment in six well plates (3 to 5 × 10^4^/well). Cells were exposed to 5 mM ascorbate in media for 1 h, washed in Dulbecco’s Phosphate Buffer Saline (PBS, 1.4 M NaCl, 27 mM KCl, 170 mM NaH_2_PO_4_, 17.6 mM KH_2_PO_4_) and re-incubated in fresh medium. Cells that received radiation were irradiated in the presence of ascorbate.

### Viability of GL261 cells

GL261 cells were collected 48 h after radiation (1, 3, 6, and 9 Gy) by trypsinization, washed in PBS, and resuspended in 1 μg/mL propidium iodide, for cell count and dye exclusion using flow cytometry using a BD FACSort (Becton Dickinson, San Jose, CA, USA). All viability assays were completed at least three times in triplicate.

### Clonogenicity of GL261 cells

Colony-forming ability was determined as described by Franken et al. (31). GL261 cells at 30–40% confluency in 100 mm dishes were treated with 6 Gy, 5 mM ascorbate or both 6 Gy and 5 mM ascorbate, trypsinized, counted, and re-plated at varying dilutions, and incubated for 12–14 days. Plates were fixed and stained in 0.5% (w/v) methylene blue solution in 50% (v/v) methanol for 1 h, and colonies consisting of at least 50 cells were counted. The surviving fraction of cells was calculated against the plating efficiency of GL261 cells for each experiment. All clonogenic assays were completed at least three times in triplicate.

### Annexin V/propidium iodide staining

Annexin V/propidium iodide staining was used to distinguish between viable cells (A^−^/PI^−^), early apoptotic cells (A^+^/PI^−^) and dead cells (A^+^/PI^+^). Cells were centrifuged at 130 × *g* at room temperature for 5 min, washed in phosphate buffered saline solution, pH 7.3 (PBS), and resuspended in Annexin V binding buffer. Small aliquots (1 × 10^6^ cells) were transferred to 1.5 mL tubes and spun at 2000 rpm for 2 min in a Biofuge fresco (Kendro, Auckland). Most supernatant was removed and 5 μL PI and 5 μL FITC-labeled AV was added to the wet pellets, which were vortexed. After 30 min in the dark on ice, 500 μL of Annexin V binding buffer was added; the cells were centrifuged for 2 min at 2000 rpm, washed once with Annexin V binding buffer, and resuspended in 1 mL of binding buffer in FACS tubes. Staining was analyzed by flow cytometry using a BD FACSort (Becton Dickinson, San Jose, CA, USA). AV/PI staining was completed three times.

### Double stranded DNA break analysis

Double stranded DNA breaks were determined at 2 and 24 h after treatments by the extent of phosphorylation of the histone variant protein, H2AX, using FITC-labeled anti-γH2AX antibodies in permeabilized cells (32). Cells were washed in PBS buffer and distributed in 96 well plates (5 × 10^5^ cells/well), washed in 200 μL FACS buffer (PBS + 1% BSA), pelleted at 2000 rpm (in a Megafuge 2.0R, Heraeus Germany centrifuge) for 2 min, and fixed in 200 μL BD Cytofix/Cytoperm solution. After 15 min incubation at 4°C, cells were washed twice in 200 μL 1× BD Perm/Wash buffer, incubated at 4°C for 15 min, pelleted and resuspended in antibody solution (50 μL anti-γH2AX antibody or isotype control diluted 1: 500 in 1× BD Perm/Wash) at 4°C for 1 h, washed twice in 1× BD Perm/Wash, and resuspended in 400 μL FACS buffer. Stained and unstained control cells were analyzed by flow cytometry.

### Intracranial mouse model

All experiments using mice were conducted in accordance with the New Zealand Animal Welfare Act 1999^1^ and were approved by the Victoria University Animal Ethics Committee. Mouse glioma GL261 cells were cultured to sub-confluence, washed in PBS, and live cells implanted into the brain of 8- to 12-week old (approx. 30 g) male C57BL/6 mice, following the procedure described by Hunn et al. (33). Briefly, animals were anesthetized by intraperitoneal (i.p.) injection of xylazine (100 mg/kg) and ketamine (10 mg/kg) (Phoenix Pharm), and Lacri-Lube (Allergan) applied across the cornea of the eye. A burr hole was drilled in the skull 0.1 mm posterior to the bregma and 2.3 mm lateral to the midline. Live cells (5 × 10^3^ in 2 μL of PBS) were administered stereotactically (Stoelting Apparatus), into the burr hole, using a Hamilton syringe with a 32-gauge needle. The needle was advanced to a depth of 2.3 mm from the brain surface and the cell suspension delivered slowly over the course of 2–3 min. Following injection, the needle was left in place for 2 min, after which time, it was raised to a depth of 1.5 mm below the brain surface and left in place for an additional 1 min. Upon withdrawal of the needle, the burr hole was sealed with bone wax and the incision sutured. Animals received sub-cutaneous analgesics [Carprofen (5 mg/kg), Norbrook Laboratories, and Buprenorphine (0.1 mg/kg), Renckitt Benckiser Pharmaceuticals], to control post-operative pain. Animals were randomly assigned into control and treatment groups (*n* = 5/group). Animals were weighed daily and humanely sacrificed by cervical dislocation when weight loss occurred (>10% of body weight) or until neurological signs of disease were evident, whichever came first.

### Whole brain irradiation of mice

**Figure 1:**
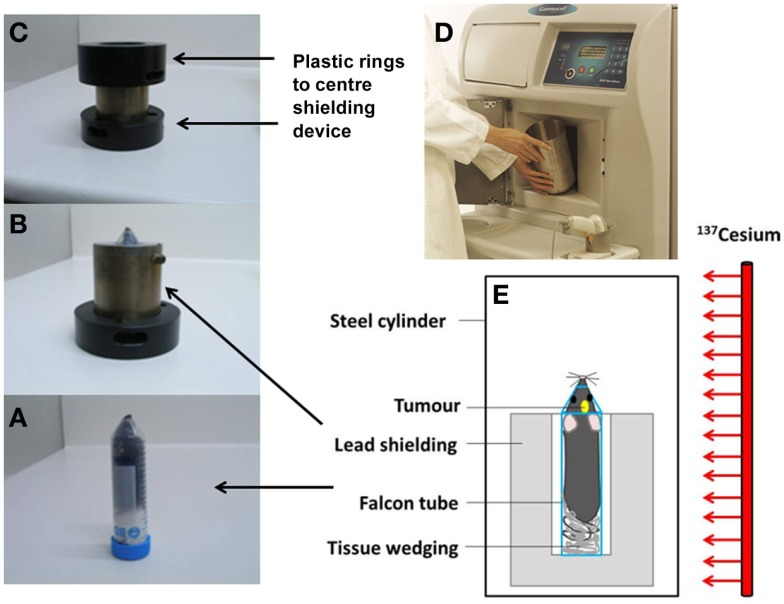
**Irradiation setup for whole brain irradiation of mice**. The mouse is positioned in a Falcon tube **(A)**, inside a 2 cm thick lead shielding device **(B)** inside plastic rings **(C)** which are placed inside the aluminum cylinder in the irradiator **(D)**. Diagram of the irradiation setup **(E)**.

Mice received a single dose of 4.5 Gy to the brain (and ≤1 Gy to the shielded body) on day 8 after surgery, based on previous work by Newcomb et al. (34). The Gammacell 3000 Elan irradiator irradiates the content of a steel cylinder with γ-rays from a sealed Cesium-137 line source (length 27 cm, diameter 1.7 cm; nominal activity 48 TBq). Tumor-bearing mice were anesthetized and placed in an upright position, stabilized by tissue paper, in a 50 mL Falcon tube without a tip to facilitate breathing. The tube was placed inside 2 cm thick custom-built lead shielding, which exposes the head to radiation while shielding the body from the ears down. The shielding was placed in the center of the steel cylinder (Figure 1).

The radiation dose received by the head and the rest of the body was verified using the thermoluminescence dosimetry system located at the Blood and Cancer Center, Wellington Hospital. The system utilizes thermoluminescent dosimeters (TLDs) based on lithium fluoride crystals doped with magnesium and titanium (“TLD-100”), in chip form with dimensions of 3.2 mm × 3.2 mm × 0.9 mm (35, 36). Prior to use, the dosimeters were reset by annealing at high temperatures in an oven (PTW-Freiburg TLDO) and sealed into plastic envelopes as rows of separate dosimeters. In order to ensure the same radiation absorption and scatter properties as during the treatment of live mice, the dosimeters were positioned between a culled mouse and a Falcon tube, along its axial length from the pelvis to the brain region. The Falcon tube including mouse and dosimeters was placed inside the lead shielding, and exposed for a set time in the gamma irradiator. The time required to deliver approximately 4.5 Gy to the mouse head using our shielding – which blocks a fraction of the radiation emitted by the source – was approximately 1.5 min. After irradiation, the TLDs underwent a pre-read anneal cycle, followed by the readout in an automatic TLD reader (Harshaw QS 5500). The calibration of the dosimeters was performed by exposing TLDs to a known radiation dose delivered by a 6 MV X-ray beam from a linear accelerator at Wellington Hospital. This dose was independently measured with a cylindrical ionization chamber following local procedures based on absorbed dose to water and calibration factors traceable to the Australian primary standard for absorbed dose.

The measurements were subject to corrections for measured variations in sensitivity for individual TLD chips, and the difference in beam energies for calibration (6 MV X-ray beam with mean energy near 2 MeV) and measurements (662 keV gamma rays for Cesium-137) (35–38). The reproducibility of the results was verified by repeating the TLD measurements for a small selection of positions in the head region and body region, finding variations not exceeding ±4%. Analysis of the results, including an estimate of the uncertainties, indicates an absorbed dose of 4.5 ± 0.3 Gy delivered to the brain region and a mean of 0.6 ± 0.04 Gy along the mouse body within the shielding. Based on these results, an acute whole body dose of 1 Sv is estimated, which is considered to be non-lethal.

### Treatment of mice with high-dose ascorbate

Tumor-bearing mice received an i.p. injection of high-dose ascorbate, 2 h prior to irradiation and after that once daily (Mon–Fri) from day 8 to 45 after surgery, at a concentration of 1 or 2 g/kg bodyweight of a sterile neutralized ascorbate solution (Sigma) in 1 mL of PBS buffer, similar to the procedure used by Chen et al. (10).

### Histological analysis of mouse brains

The auto-fluorescent property of H&E stained blood was used to quantify the amount of intravascular and extravascular blood in each section. An Olympus 1 × 51 inverted microscope and Olympus DP72 camera were used to take fluorescent images of the entire tumor at 4× using a mercury bulb and a band-pass filter to measure wavelengths between 604 and 644 nm. ImageJ 1.48v^2^ was used to measure the percentage of blood in the tumor. Fluorescent images were converted into 8-bit grayscale images. The total area of the tumor was measured by tracing around the tumor border with the freehand selection tool. The threshold tool was adjusted to select and measure only the auto-fluorescent blood in the image. The following formula was used to convert these measurements into the percentage of blood in the tumor:

$$\text{percentage of tumor consisting of blood}=\frac{\text{area of blood}}{\text{area of tumor}}\times 100$$

Necrosis was measured using brightfield images of the same H&E stained sections. Areas of necrosis were traced around and measured with the freehand selection tool. The following formula was used to convert these measurements into the percentage of necrosis in the tumor:

$$\text{percentage of tumor consisting of necrosis}=\frac{\text{area of necrosis}}{\text{area of tumor}}\times 100$$

### Statistical analysis

Statistical significance of differences in 48 h survival and clonogenicity of GL261 cells between different treatments were calculated using unpaired two-tailed student *t* tests (Microsoft v 2010; Redmond Campus, Redmond, Washington, DC, USA). The Mantel–Cox log-rank test (Prism 5.0 Graph Pad Software, Inc., La Jolla, CA, USA) was performed to determine the statistical significance between Kaplan–Meier survival curves. In all instances, *p* < 0.05 was considered statistically significant.

## Results

### Sensitivity of GL261 cells to radiation, ascorbate, and combined treatments

**Figure 2:**
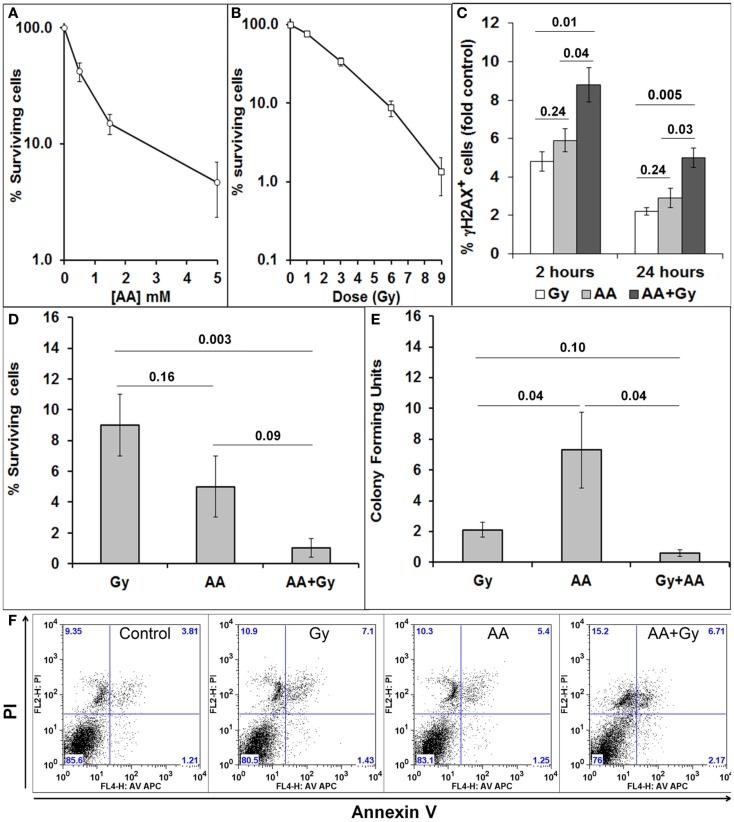
**Sensitivity of GL261 to radiation and ascorbate treatments**. **(A)** Cell survival 48 h after a 1 h exposure to different doses of ascorbate (AA), measured by PI exclusion and flow cytometry; **(B)** cell survival 5 days after irradiation with 1, 3, 6, and 9 Gy, measured by PI exclusion and flow cytometry; **(C)** DNA damage and repair as fold change (compared to control) of % cells with DSBs was measured as the extent of H2AX phosphorylation (fluorescence intensity) using flow cytometry and anti-γH2AX antibodies at 2 and 24 h after treatments; **(D)** Cell survival 48 h after 6 Gy, 5 mM AA, or combined treatment as measured by PI exclusion and flow cytometry; **(E)** clonogenicity (colony-forming units) 10–12 days after 6 Gy, 5 mM AA, or combined treatment. Colonies of 50 cells or more were visualized by methylene blue staining, counted, and numbers are presented as percentage of untreated controls. **(F)** Lack of apoptosis of GL261 cells 48 h after 6 Gy, 5 mM AA, or combined treatment as measured by FACs analysis of AV/PI staining. Values are averages ± SEM of at least three different experiments conducted in triplicate. Numbers above bars denote *p* values (unpaired two-tailed student *t*-test).

GL261 is a mouse glioma cell line, which forms tumors in immunocompetent C57BL/6 mice. We first tested the sensitivity of GL261 cells *in vitro* to a single exposure of different doses of radiation (Gy) (Figure 2A) and a 1 h exposure to different concentrations of ascorbate (AA) (Figure 2B). We have previously shown that our GBM cell lines do not produce many DSB after a single exposure of 1 or 3 Gy. All our *in vitro* research therefore has used a single dose of 6 Gy to determine the effect of a radiation alone and in combination with 5 mM ascorbate on viability, clonogenicity, generation, and repair of DSBs and cell cycle progression (7, 8). Concentrations of 5 mM ascorbate have been used by a number of authors in the field [reviewed in Ref. (1)]. Similar to our previous research with a panel of GBM cell lines (7, 8), GL261 cells sustained a substantial amount of DNA damage, as shown by the percentage of cells with DSBs 2 h after treatments. Initial DNA damage as well as DNA damage repair was significantly worse after combined treatment compared with single treatments (Figure 2C). We determined the percentage of GL261 cell survival after 48 h (Figure 2D) and colony-forming ability after 10–12 days (Figure 2E), in response to both single and combined treatments. Combining both treatments decreased 48 h survival and clonogenicity compared with radiation treatment alone. GBM cells are very resistant to apoptosis (7, 39). GL261 cells also did not undergo apoptosis when exposed to single and combined treatments *in vitro* (Figure 2F).

### Effect of radiation, ascorbate, and combined treatments on mouse survival

**Figure 3:**
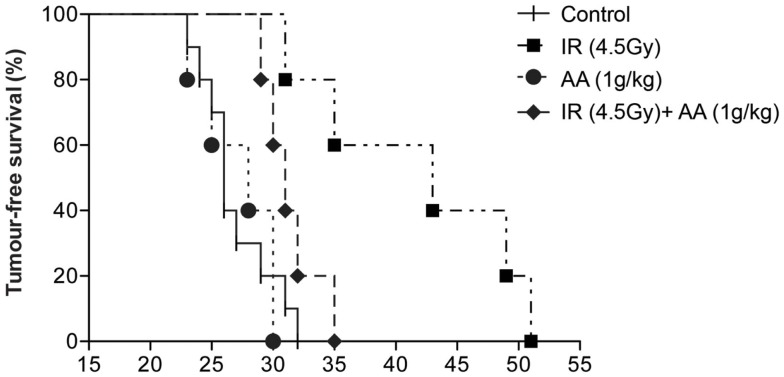
**Effect of a single dose of radiation, daily i.p. ascorbate and combined treatment on survival of tumor-bearing C57BL/6 mice**. Tumor-bearing C57BL/6 mice were divided into four groups: one group received a single dose of 4.5 Gy to the brain 8 days after tumor implantation, a second group received daily intraperitoneal injections of ascorbate (day 8–45) after implantation, a third group received both treatments, and a fourth control group received no treatment. Animals were sacrificed when they had lost 10% of their weight in 24 h or after neurological symptoms were evident. Results are representative of four experiments (two with 1 g/kg ascorbate and two with 2 g/kg ascorbate).

Our next step was to validate the *in vitro* radio-sensitizing effect of high-dose ascorbate on GL261 clonogenicity in an intracranial mouse glioma model. Live GL261 cells (5 × 10^3^ in 2 μL of PBS) were implanted into the brain of 8- to 12-week-old (approximately 30 g) male C57BL/6 mice. Tumor-bearing mice were divided into four groups: one group received a single dose of 4.5 Gy to the whole brain [based on previous work by Newcomb et al. (34)] 8 days after tumor implantation, a second group received daily i.p. injections of ascorbate (1 g/kg AA) from day 8 to 45 after implantation [based on previous work by Chen et al. (10)], a third group received both treatments, and a fourth control group received no treatment at all. Tumor-bearing mice were culled when weight loss due to tumor growth exceeded 10% of body weight or neurological symptoms of disease (40), and survival of animals from all groups are shown in Figure 3. Median survival for untreated animals survived was 26 days (range 23–32). Daily doses of intraperitoneal ascorbate did not affect survival (median 28 days; range 23–30). A single dose of radiation increased survival of tumor-bearing mice significantly (median 43 days; range 31–51; *p* = 0.002) compared with untreated control animals. However, in sharp contrast with our *in vitro* data, tumor-bearing mice receiving both a single dose of radiation and daily i.p. ascorbate needed to be culled significantly sooner (median 31 days; range 29–35) than animals treated with a single dose of radiation alone (*p* = 0.0275). These data suggest that rather than making the tumor more sensitive to radiation, ascorbate protected the tumor from radiation damage. We obtained very similar results when injecting 2 g/kg i.p. ascorbate daily (results not shown).

### Effect of radiation, ascorbate, and combined treatments on tumor histology

**Figure 4:**
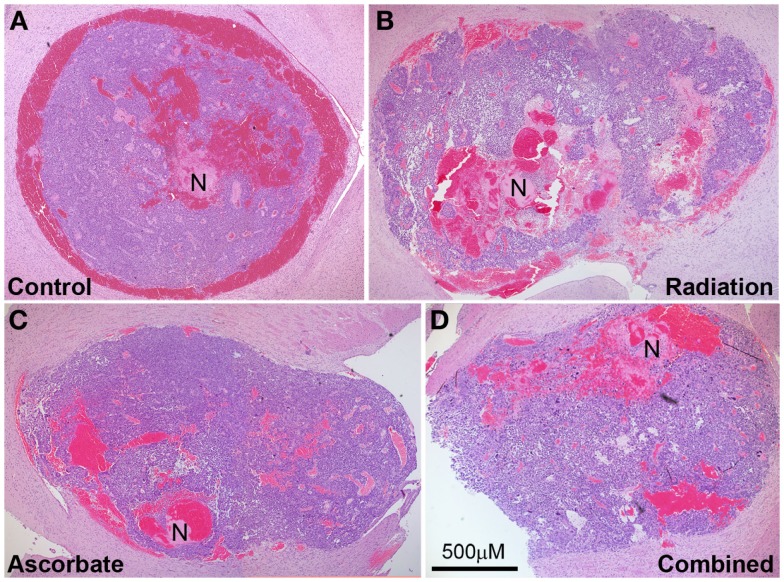
**Representative tumor histology of mice that were not treated (A), treated with a single dose of 4.5 Gy (B), daily i.p ascorbate (C), and both treatments (D)**. Analysis of necrosis and presence of blood in tumors was carried out by H&E staining at multiple sections across the tumors of several mice. N indicates necrosis. Magnification 4×; bar represents scale for all four photos (500 μM).

**Figure 5:**
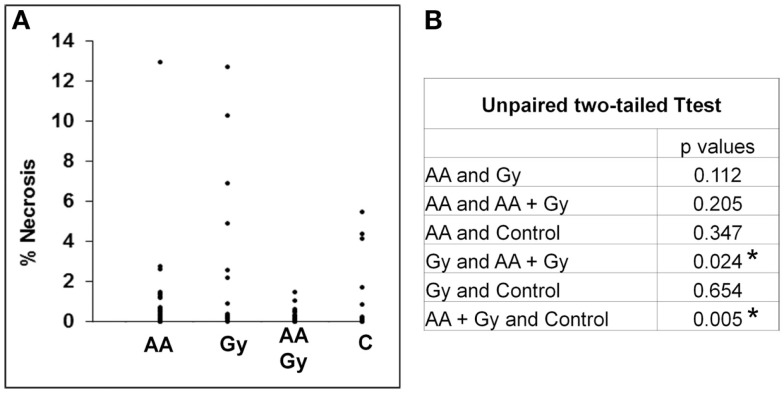
**Percentage necrosis (A) and statistical analysis (B) of tumors treated with daily i.p. ascorbate (AA), a single dose of radiation (Gy), combined treatment (AA + Gy), and no treatment (C)**. *p < 0.05 is statistically significant.

Tumor-bearing brains were collected from animals at the endpoint of the experiment, which was weight loss of >10% or neurological symptoms of disease. Tumors were approximately the same size upon collection but irradiated only mice took longer to develop these tumors compared with control mice, ascorbate treated mice, and mice treated with both radiation and ascorbate (see section above). Analysis of necrosis and presence of blood in tumors was carried out by H&E staining at multiple sections across the tumors of at least four mice (a representative example is shown in Figure 4). GL261 tumors contained substantial amounts of intravascular and extravascular blood, regardless of whether they were treated or not (Figure 5A). Untreated tumors had variable but low levels of necrosis, making up 0–6% of tumor area. Radiation treatment doubled the incidence of necrosis, up to 12% of tumor area. Treatment with ascorbate alone had no effect on necrosis. Strikingly, addition of ascorbate to radiation prevented the radiation-induced necrosis, even below the level of necrosis in control tumors (Figure 5B). These results were consistent with a radio-protective effect of ascorbate on the tumor, resulting in shorter survival times of tumor-bearing mice receiving both ascorbate and radiation. Fluorescent confocal microscopy using anti-PCNA (Proliferating Cell Nuclear Antigen) antibodies showed a complete lack of proliferation in the normal brain tissues and proliferation in 45–50% of cells throughout the tumors, regardless of whether the mice had been treated or not (results not shown).

## Discussion

We set out to validate our previous reports on the *in vitro* radiosensitizing effect of high-dose ascorbate in primary GBM cells (7, 8) in an intracranial mouse glioma model. Although high-dose ascorbate made GL261 cells more sensitive to radiation *in vitro*, unexpectedly it protected tumors derived from GL261 cells implanted in the brain of mice from radiation injury. To our knowledge, this is the first time the combination of high-dose ascorbate with radiation has been tested in mice. Previous studies have shown that high-dose ascorbate as a single agent (two daily i.p. injections of 4 g/kg) significantly retarded tumor growth of rat 9L gliosarcoma cells implanted subcutaneously in the flank of immunocompromised mice (10). In contrast, our immunocompetent mice with brain tumors treated with 1 or 2 g/kg daily i.p. ascorbate as a single agent developed tumors at the same time as untreated animals. While it may have been useful to examine the 4 g/kg doses used in other studies, our tumor-bearing mice did not cope with the osmotic challenge of buffered ascorbate sodium salt at doses in excess of 2 g/kg, preventing the escalation to 4 g/kg.

We hypothesize that the discrepancies between the *in vitro* data, the mouse subcutaneous and intracranial data can be explained by differences in tumor microenvironments. The DNA damage and other anti-tumor effects of ascorbate require the extracellular conversion to hydrogen peroxide [reviewed in Ref. (1)]. Laboratory conditions are characterized by optimal access to oxygen, nutrients, and space in the absence of normal stroma, and extracellular ascorbate is readily converted to peroxide in this setting. Although the subcutaneous microenvironment of “flank models” mimics a “real” tumor with access to oxygen, nutrients, growth factors, and cytokines available in the systemic blood circulation, it does not model the conditions in the intracranial interstitial fluid inside the blood–brain barrier, or the highly specialized interactions between glioma cells on one hand and astrocytes and microglial cells on the other hand that may control the fate of ascorbate in the brain tumor microenvironment.

Astrocytes are a subset of glial cells responsible for maintaining intracranial homeostasis by regulating cerebral blood flow, neuronal activity through neuron–astrocyte metabolic coupling and protect neurons from toxic wastes and chemotherapeutic drugs (41, 42). Small, early stage tumors that develop in this microenvironment can harness the protective effects of astrocytes as shown by Kim and colleagues, who reported that brain metastases from NCI-H358 lung cancer cells were less sensitive to rapamycin-induced apoptosis when co-cultured with astrocytes but not when co-cultured with fibroblasts (41). These authors also showed that co-culture of human MDA-MB-231 breast cancer cells with murine astrocytes protected the cancer cells from apoptosis by vincristine through upregulation of the anti-apoptotic survival genes, BCL2L1, TWIST1, and GSTA5. Protection of tumor cells by astrocytes was mediated by continued physical contact between astrocytes and tumor cells and direct communication via GAP junctions (42). Strong expression of these survival genes was also seen in clinical specimen from breast cancer brain metastases but not in breast cancer metastasized to the lungs (42). With respect to radiation sensitivity, human NSC11 stem cell-like GBM cells grown *in vitro* were reported to be much more sensitive to radiation than the same cells implanted in mouse brains (43, 44).

The brain microenvironment is also unique with respect to ascorbate metabolism. Ascorbate passes through the blood–brain barrier very slowly but it is actively transported into the cerebrospinal fluid via the high affinity sodium-dependent vitamin C transporter, SVCT2. Ependymal cells that line the choroid plexi and produce cerebrospinal fluid from blood plasma highly express SVCT2 (3, 45–47). Neurons further concentrate intracellular ascorbate to millimolar levels (3, 45, 47), where it acts as a free radicals scavenger (3), assists the cellular hypoxia response through hydroxylation of hypoxia-inducible factor 1α (48), promotes collagen synthesis, and neuronal maturation and transmission (3). Therefore, ascorbate concentrations in the brain are very high (2–10 mM) compared with liver (0.8–1 mM), muscle (400 μM), cerebrospinal fluid and interstitial fluid (160–400 μM), and blood plasma (40–60 μM) (3). These high ascorbate concentrations in the brain are generated through active transport via the high affinity sodium-dependent vitamin C transporter, SVCT2, which is highly expressed on ependymal cells and neurons (3, 45). So, rather than through the blood–brain barrier, ascorbate is actively transported into the cerebrospinal fluid through SVCT2.

Astrocytes and microglial cells (central nervous system resident macrophages) do not normally express SVCT2 but acquire ascorbate in its oxidized form, dehydroascorbate (DHA), imported via the glucose transporter, GLUT1. Once inside the cell DHA is rapidly reduced to ascorbate, resulting in intracellular ascorbate levels in astrocytes and microglial cells similar to those of cerebrospinal and interstitial fluid of 160–400 μM, adding to their anti-oxidant capacity (3, 45).

The mechanism of ascorbate uptake in gliomas and GBM is likely to include both SVCT2 and GLUT1. A recent report suggests that proliferative neural progenitor cells up-regulate SVCT2 in response to ascorbate (49), a mechanism that could also transport ascorbate into GBMs. An extensive analysis of gene expression and DNA copy number in gliomas (50–52) has indicated the SVCT2 locus is amplified in gliomas, and expression is significantly higher than in normal brain tissue. However, data from other expression studies suggest that SVCT2 expression in gliomas may be lost (53, 54). Further, gene expression does not guarantee transporter activity. Rodriguez and colleagues showed that the high SVCT2 expression in the TC620 oligodendroglioma cell line was offset by its lack of activity and intracellular location (55). *In vivo*, the acidic tumor environment is likely to reduce the activity of the SVCT2 transporters, which have an optimal activity at a slightly alkaline pH (56). For gliomas that lack an active SVCT2, tumor associated microglial cells, which can make up to 30% of tumor mass in high grade gliomas could supply the glioma cells with DHA taken up via GLUT1 (57).

Regardless of the mechanism of uptake, glioma cells may be able to rapidly acquire significant quantities of ascorbate. This will have two effects – first, it will prevent the extracellular conversion of ascorbate to hydrogen peroxide, neutralizing the DNA damage potential. Second, the anti-oxidant activity of ascorbate will protect the cell from radiation-induced oxidative damage. Taken together, our results strongly suggest that the unique brain microenvironment promotes accumulation of ascorbate inside GBM cells to concentrations high enough to facilitate radioprotection. This is in sharp contrast with the reported pro-oxidant effect of extracellular ascorbate *in vitro* and in flank xenograft models [reviewed in Ref. (1)].

Although our orthotopic glioma model mimics the brain microenvironment and is therefore clinically more relevant than subcutaneous flank models, it has certain limitations that make extrapolation to the clinical setting problematic. GL261 cells represent a stage II–III glioma and not a stage IV GBM, form well-defined tumors rather than the diffuse pattern often seen in GBMs and are a lot more sensitive to radiation and ascorbate than true primary and established GBM cell lines as shown previously (7). Perhaps more importantly, our mice received only a single dose of 4.5 Gy, whereas standard radiation therapy for GBM patients consists of daily fractions of 2 Gy over 10 weeks to a total dose of 60 Gy (24, 58). In addition, most GBM patients will receive the chemotherapy drug temozolomide at the same time as radiation therapy as well as in the adjuvant setting in the form of several cycles after completion of radiation therapy (24). We also did not measure tissue ascorbate levels in this study, which should be done in future studies to determine to what extent the injected ascorbate affects ascorbate levels in both brain and tumor tissue. However, although our findings cannot be directly extrapolated to the clinical setting, the underlying problem with combining radiation and high-dose ascorbate remains the same: does the ascorbate get into the glioma cells and act as an anti-oxidant, protecting the tumor from radiation OR does it remain outside the cells, generating hydrogen peroxide and acting as a pro-oxidant. It may be useful in this context to test individual patient tumors for the presence and activity of SVCT2.

In contrast to combining high-dose ascorbate with radiation in brain cancers, clinical studies combining high-dose ascorbate with chemotherapeutic agents for metastatic ovarian and pancreatic cancer have produced more promising results. Adding high-dose ascorbate to existing chemotherapy regimens decreased chemotherapy-related toxicities and increased patient overall survival even if the latter was not statistically significant in these small phase I trials (11, 22, 23). It is important to note, however, that these clinical studies were not done in patients with brain cancers, and included drugs that do not work primarily by generating free radicals. Ascorbate would not have affected the efficacy of these chemotherapy drugs whether it acted as a pro-oxidant or an anti-oxidant.

## Author Contributions

Carole Grasso, Marie-Sophie Fabre, and Cameron S. Field conducted and analyzed the intracranial mouse experiments; M. Leticia Castro conducted and analyzed *in vitro* GL261 experiments; Sarah V. Collis performed the histological analysis. Nanette Schleich designed shielding device and performed the dosimetry. Melanie J. McConnell contributed to experimental design and data interpretation. Patries M. Herst developed the concept and contributed to the design of the lead shielding device, experiments, and data analysis. All authors contributed to writing of and approved of the final version of the manuscript.

## Conflict of Interest Statement

The authors declare that the research was conducted in the absence of any commercial or financial relationships that could be construed as a potential conflict of interest.

## Footnotes

^1^www.legislation.govt.nz/act/public/1999/0142/latest/DLM49664.html

^2^http://imagej.nih.gov/ij/index.html
